# Current Topics in Dermatophyte Classification and Clinical Diagnosis

**DOI:** 10.3390/pathogens11090957

**Published:** 2022-08-23

**Authors:** Alex E. Moskaluk, Sue VandeWoude

**Affiliations:** Department of Microbiology, Immunology, and Pathology, Colorado State University, Fort Collins, CO 80523, USA

**Keywords:** dermatophytes, diagnostics, *Microsporum canis*, mycology, ringworm, skin disease

## Abstract

Dermatophytes are highly infectious fungi that cause superficial infections in keratinized tissues in humans and animals. This group of fungi is defined by their ability to digest keratin and encompasses a wide range of species. Classification of many of these species has recently changed due to genetic analysis, potentially affecting clinical diagnosis and disease management. In this review, we discuss dermatophyte classification including name changes for medically important species, current and potential diagnostic techniques for detecting dermatophytes, and an in-depth review of *Microsporum canis*, a prevalent zoonotic dermatophyte. Fungal culture is still considered the “gold standard” for diagnosing dermatophytosis; however, modern molecular assays have overcome the main disadvantages of culture, allowing for tandem use with cultures. Further investigation into novel molecular assays for dermatophytosis is critical, especially for high-density populations where rapid diagnosis is essential for outbreak prevention. A frequently encountered dermatophyte in clinical settings is *M. canis*, which causes dermatophytosis in humans and cats. *M. canis* is adapting to its primary host (cats) as one of its mating types (MAT1-2) appears to be going extinct, leading to a loss of sexual reproduction. Investigating *M. canis* strains around the world can help elucidate the evolutionary trajectory of this fungi.

## 1. Introduction

Dermatophytes are the most ubiquitous fungal pathogens worldwide, responsible for a majority of skin and nail infections [1,2,3]. Globally, the estimated lifetime risk of developing dermatophytosis is 10–20%, with infections of the feet being the most common [4,5]. Given the high infection rates, treating dermatophytes globally costs $500 million annually [6]. An accurate diagnosis is key for patient management and the implementation of appropriate therapies. Many forms of dermatophytosis can be difficult to clinically distinguish from other skin ailments as clinical presentation overlaps between the diseases [7]. Additionally, a significant portion of dermatophyte cases occur in developing countries, where populations are unable to access health care [8,9]. Misdiagnosis can result in dangerous consequences, particularly in immunocompromised patients, as disease can progress to deeper invasion, leading to disseminated dermatophytosis and invasive dermatitis [10,11,12,13]. Misdiagnosis can also occur due to the misidentification of the dermatophyte responsible for the infection. As with other fields in mycology, medical mycology has recently experienced reclassification of various species as the use of genetics has become more prevalent in defining species [14,15]. Name changing has affected many genera of dermatophytes, which can impact clinical databases and certain diagnostic techniques. In this review, we discuss (1) dermatophyte classification with regard to human and animal infections, (2) diagnostic techniques currently used for detecting dermatophytes in clinical settings, (3) potential future directions for dermatophytosis diagnostics, and (4) a comprehensive clinical review on prevalent zoophile *Microsporum canis*. We focused on *M. canis* as it frequently infects both humans and animals, making it one of the most encountered dermatophytes in clinical settings [16,17].

## 2. Introduction to Dermatophytes

Dermatophytes are a classification of fungi that invade and degrade keratinized tissues including hair, skin, nails, and feathers [18]. These fungi belong to the Ascomycota phylum, Eurotiomycetes class, Onygenales order, and Arthrodermataceae family [15,19]. There are currently seven accepted genera of dermatophytes: *Trichophyton*, *Epidermophyton*, *Nannizzia*, *Paraphyton*, *Lophophyton*, *Microsporum,* and *Arthroderma* [15]. As with other fungi families, names of species have continuously changed as the field of mycology has switched from naming based on morphology and clinical disease of isolates to including a molecular approach [14,15]. Additionally, the sexual form (teleomorph) and asexual form (anamorph) used to be classified as two separate species with different names [16]. Recently, names of teleomorphs and anamorphs have been consolidated, resulting in the “One Fungus = One Name” system for species’ identification [20,21]. Further categorization of dermatophytes places species into three different groups based on their habitat: anthropophilic (living on humans), zoophilic (living on animals), and geophilic (living in the environment) [14,15,22]. As mycological naming systems have continued to evolve, molecular characterization has been implemented in conjunction with other approaches to better define dermatophyte species.

### 2.1. Molecular Characterization of Dermatophytes

Molecular approaches have been applied to dermatophytes to assist in classification and epidemiological studies. Dermatophyte genomes range from 2.25 Mb to 24.1 Mb, and the full genomes of several species, including *Microsporum canis,* have been annotated [23]. Dermatophyte genomes are haploid and contain relatively little repetitive DNA [24]. Conidia (spores) have a single nucleus and hyphae generally are multinucleated cells with genetically unique nuclei [25]. The genomes of dermatophyte species are relatively conserved, with over 6000 orthologs shared among anthropophiles, zoophiles, and geophiles [23]. *M. canis* has 943 unique genes, the most heterogeneity characterized to date among dermatophytes [23]. Using two gene regions (internal transcriber spacer region ITS and partial β-tubulin), Baert et al. recently proposed a new classification scheme for dermatophyte species using a phylogenetic approach [22]. This resulted in numerous species being reclassified, expanding the *Nannizzia*, *Paraphyton*, *Lophophyton,* and *Trichophyton* genera while condensing the *Arthroderma* and *Microsporum* genera [22]. This included renaming the clinically important species *Nannizzia persicolor* (former name *Arthroderma persicolor*), *Nannizzia nana* (former name *Microsporum nanum*), *Trichophyton mentagrophytes* (former name *Arthroderma vanbreuseghemii*), and *Nannizzia gypsea* (former name *Microsporum gypseum*) [22]. This reclassification also shifted the percentage of species in a genus that are anthropophiles, zoophiles, and geophiles [22], making it more critical to identify down to the species level, instead of genus, in clinical infections.

In addition to genus’ and species’ classification, epidemiological studies have been conducted to characterize dermatophyte genetic variations, evaluating DNA sequences of mitochondria [26], non-transcribed spacer regions (rDNA) [27], random amplified polymorphic DNA [28,29], microsatellites [30,31,32], and RNA sequencing [33]. Microsatellite DNA polymorphisms have been identified in dermatophytes [30,31], providing a technique to rapidly characterize strain variation at low cost and offering a useful method for genotypic comparisons among large sample sets. Several studies have implemented this approach for *M. canis* sample sets from various regions in the world, demonstrating intraspecies genetic differences [30,31,32,34,35,36,37]. Genetic understanding of dermatophytes has greatly increased in recent years, allowing for a better comprehension of basic dermatophyte processes including how infections begin.

### 2.2. Initiation of Dermatophyte Infections

Dermatophytes are free-living in the environment, but under certain conditions can cause infections in humans and animals. These fungi are septate, hyaline, filamentous molds that can produce spores (conidia) and are mainly composed of mycelium [38]. Mycelium structures are formed from the amalgamation of fungal tubular structures known as hyphae [38]. Mycelium performs the physiologic functions of nutrient absorption, spore creation, and environmental sensing of light, temperature, and nutrients [38,39,40,41]. Different types of conidia are formed depending on the dermatophyte species and environmental conditions [38]. For example, asexual spores can form as macroconidia (large, multi-septate conidia), microconidia (small, unicellular conidia), and arthroconidia (infectious fragments of hyphae) [38].

**Figure 1:**
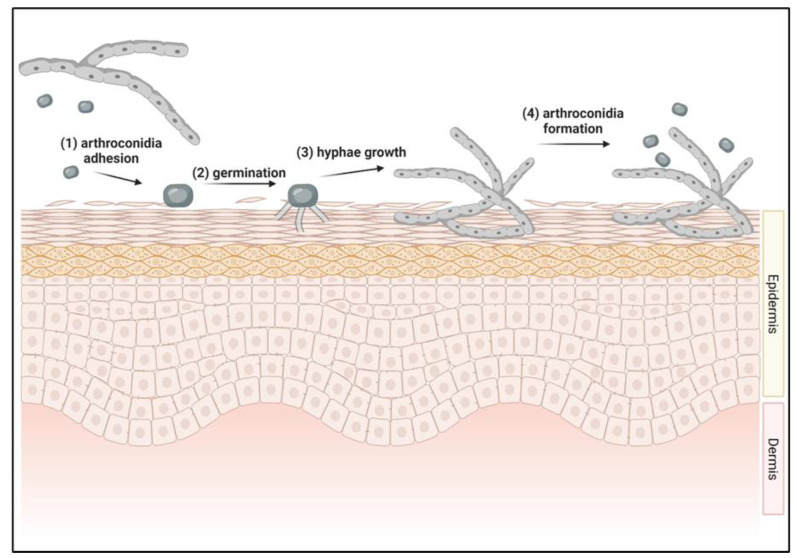
Initiation of dermatophyte infection in skin. **(1)** Arthroconidia from environment or other infected host contacts new host’s skin. Adhesion to skin occurs between 2–6 h after contact. **(2)** Arthroconidia begins to germinate in the top layer of the epidermis, forming germ tubes. **(3)** Hyphae continue to grow within the epidermis. **(4)** Within 7 days of infection, arthroconidia are formed, allowing for the cycle to repeat.

The initiation of a dermatophyte infection begins when infectious portions of dermatophytes called arthroconidia adhere to keratinized tissues [42,43,44] (Figure 1). Arthroconidia first adhere to the epidermis within 2 to 6 h after contact and begin to germinate in the stratum corneum [42,43,44] (Figure 1). As arthroconidia begin to germinate, these spores develop germ tubes that can penetrate the first layer of the epidermis, the stratum corneum [44] (Figure 1). The pH at the site of infection becomes more basic as the dermatophyte degrades keratin, aiding in the activity of downstream fungal proteases [45]. Fungal hyphae continue to grow and invade keratinized tissues and begin producing arthroconidia within 7 days of infection, allowing the fungus to spread to other anatomical locations of the original host, to other hosts, or to contaminate the environment [46] (Figure 1).

Arthroconidia cannot invade healthy tissue because the host’s immune system prevents the fungi from infecting healthy epidermis [2,18,47,48,49]. Therefore, predisposing factors are typically present for an infection to occur. Common predisposing factors for infection are young age, immunosuppression, nutritional deficiency, skin trauma, and high environmental temperature and/or humidity [2,16,18,48,49]. One study demonstrated that cats experimentally exposed to dermatophyte spores remained uninfected unless a predisposing factor was induced; factors in this study were occlusive bandage and restricted grooming [50]. The unique metabolic pathways of dermatophytes that facilitate keratin invasion and digestion are potential sites for novel therapeutic development and intervention.

### 2.3. Dermatophyte Viability

While infections can result from direct transmission from an infected animal, dermatophytosis can also result following contact with viable environmental conidia. Environmental contamination with arthroconidia is very common in places where infected individuals reside such as animal shelters or animal raising establishments [51,52,53]. High exposure locations such as swimming pools, nails salons, and wrestling mats can also lead to dermatophytosis from environmental contamination [54,55,56]. While the length of infectivity of arthroconidia in the environment for inducing dermatophytosis in a host is uncertain, under laboratory conditions, arthroconidia have remained viable for up to 4.5 years, depending on the dermatophyte species [57,58,59,60]. Long-term viability is critical for the laboratory analysis of isolates, particularly of rare dermatophyte species [61,62]. Long-term preservation methods for dermatophytes include lyophilization and cryopreservation using liquid nitrogen vapor, storage at low temperatures (−20 °C to −70 °C), and commercial cryopreservation kits [61,63,64,65,66,67], with commercial kits used most successfully (Microbank; Pro-Labs Diagnostics, Richmond Hill, Ontario, Canada). These protocols harvest mycelium and conidia that are subsequently stored at −80 °C on porous beads suspended in a cryopreservative fluid, allowing recovery over a 2-year period for a variety of dermatophyte species [61]. Understanding the viability of arthroconidia in clinical and laboratory settings can help improve decontamination protocols, reduce potential outbreaks, and assist in maintaining bioarchives for future studies.

## 3. Dermatophyte Classification

Dermatophytes are broadly classified into three groups depending on their environmental habitat and include anthropophiles (living on humans), zoophiles (living on animals), and geophiles (living in the soil) [14,15,22]. The distinction between these groups can be blurred as species can become adapted to certain hosts, switching their preferred habitat [14,16,68,69,70]. Group classification is important as clinical presentation can be influenced by the type of dermatophyte causing the infection [15,24]. Over 40 species from all three classifications have the potential to cause infections in humans [14,24]. The following subsections examine habitat classification groups and the type of infections caused by the most clinically relevant species.

### 3.1. Anthropophilic Dermatophytes

Dermatophytes that preferentially infect humans are classified as anthropophilic dermatophytes that theoretically evolved from geophilic dermatophytes [14,68,69]. Animals can sometime be infected with anthropophilic dermatophytes following anthropo–zoonotic transmission [71,72]. Approximately 10 dermatophyte species belong to this group with the main genera including *Trichophyton* and *Epidermophyton* [15]. The majority of infections are caused by *Trichophyton rubrum*, *Trichophyton interdigitale*, and *Epidermophyton floccosum*, with *T. rubrum* being the most widely spread dermatophyte that infects humans [73,74]. These species are well adapted to the human physiology and immune system, resulting in a dampened immune response and mild clinical signs [24]. Occasionally, non-inflammatory, chronic infections with more significant clinical features can last months to years [15]. Only one mating type has been documented per species, suggesting that they rely exclusively on asexual reproduction [15,75,76]. It has been theorized that since these species have adapted to humans, they face less selective pressure, resulting in the loss of one mating type [77].

**Figure 2:**
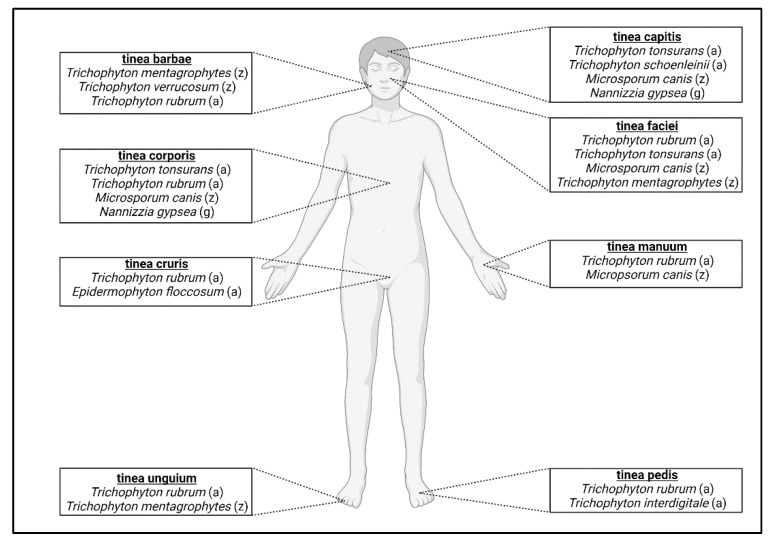
Classification of dermatophytosis in humans. Anatomic location of infection determines the type of dermatophytosis. For each classification, the most prevalent fungal species that cause infection are listed; (a) = anthropophile; (z) = zoophile; (g) = geophile.

As anthropophiles have become adapted to humans, species have developed preferences for specific locations on the body. For example, infection of the feet is called tinea pedis (“athlete’s foot”) [24,78]. Other forms of localized dermatophytosis include tinea capitis (infection of the scalp), tinea unguium (infection of nails), tinea barbae (infection of the beard), tinea faciei (infection of the face), tinea corporis (infection of the body), tinea manuum (infection of the hands), and tinea cruris (infection of the groin region) [18] (Figure 2). *T. rubrum* is the most prevalent dermatophyte infecting humans worldwide and is responsible for the majority of tinea pedis cases [1,24,25]. *T. interdigitale* also causes tinea pedis and is a clonal offshoot of *T. mentagrophytes* [15,74]. *T. tonsurans* is one of the primary agents responsible for tinea capitis infections around the world [24,79] (Figure 2, Table 1). A rare form of tinea capitis called tinea capitis favosa is caused by *Trichophyton schoenleinii* and usually occurs in children and adolescents [74,80,81]. The distribution of these infections varies according to geographic and socioeconomic factors, with tinea pedis occurring more frequently in developed countries and tinea capitis in developing countries [23]. Age also plays a role in which type of dermatophytosis is present, as older patients tend to have tinea unguium, whereas children more frequently acquire tinea capitis [82]. Tinea unguium can also be referred to as onychomycosis, which encompasses all fungal infections of the nail [83]. Most of these infections are due to *T. rubrum* and zoophile *Trichophyton mentagrophytes* [18]. Tinea barbae infections are mostly caused by zoophilic dermatophytes, *T. mentagrophytes* and *T. verrucosum*, and anthropophilic *T. rubrum* [68,84,85]. The dermatophytes responsible for tinea corporis infections can depend on the route of transmission [86]. Human-to-human transmission infections are generally due to *T. rubrum* and *T. tonsurans*, whereas contact with animals can lead to infection from *M. canis* [86]. Tinea faciei is considered a special form of tinea corporis and is usually caused by *T. rubrum*, *T. mentagrophytes*, *T. tonsurans*, or *M. canis* [87,88,89] (Table 1). *T. rubrum* and *E. floccosum* are responsible for the majority of tinea cruris (“jock itch”) infections [18]. Tinea manuum infections are caused mostly by *T. rubrum* or *M. canis* and is usually associated with tinea pedis infections [18,90,91] (Figure 2, Table 1). While the majority of dermatophytosis cases in humans are due to anthropophiles, zoophiles can also cause infections in humans and zoonotic dermatophyte infections can also occur (further discussed below).

Recently, a new anthropophilic species (*Trichophyton indotineae*) has become widespread, causing recurrent infections where some isolates are resistant to terbinafine treatment [101,102]. This fungus can cause various forms of dermatophytosis including tinea pedis, tinea unguium, tinea cruris, tinea corporis, and tinea faciei [102,103]. Current treatment involves other anti-fungal medications such as itraconazole; however, strains from Germany have been shown to have reduced sensitivity to this drug [102,104]. As this species continues to spread to more countries, it is important for health care workers to be aware of this dermatophyte and its potential resistance to therapies.

### 3.2. Zoophilic Dermatophytes

Zoophilic dermatophyte species have evolved to live on non-human animals [16]. The main species that cause infections in animals are *Microsporum canis*, *Nannizzia persicolor*, *Nannizzia nana*, *Trichophyton equinum*, *Trichophyton mentagrophytes*, and *Trichophyton verrucosum* [16]. In humans, the infections are mostly caused by *M. canis*, *T. mentagrophytes*, and *T. verrucosum* [96]. When these infections occur in humans, there is usually significantly more inflammation and there is a shorter course of infection than those noted for anthropophilic dermatophytosis infections [15]. It is thought that the more robust inflammatory response may be attributed to a lack of host–pathogen adaptation. As a corollary to this relationship, these fungi can also undergo sexual reproduction [75,105] as two mating types exist in nature and can reproduce when two isolates of opposite types encounter each other [15,75,76]. However, for many of these species, it has been shown that the proportion of the two mating types has become unequal, leading to increasingly more reliance on asexual reproduction [75]. Zoophiles that infect soil-dwelling animals are more likely to have both mating type isolates and undergo sexual reproduction than zoophiles from non-soil-associated animals [70]. As these fungal species become more adapted to a particular host species, infections tend to become more reliant on asexual reproduction as noted in human infections. Dermatophytosis occurs more often in mammals than in reptiles and birds [106,107,108].

Dermatophytosis, or ringworm, in animals is generally not further classified based on infection location as is standard for human infections. Clinical signs typically include circular alopecic lesions with erythematous margins, and pruritus is not commonly observed [99]. Animals can be asymptomatic carriers of dermatophytes, resulting in occult transmission to other animals or humans in close contact [99].

Cats are the primary host for *M. canis* and will be explored further in later sections. Other zoophiles infect livestock such as pigs and ruminants, putting farmers and livestock handlers at higher risk for zoonotic infection [19,53]. The most frequently isolated dermatophyte species on ruminants is *Trichophyton verrucosum* [98] (Table 1). An increase in dermatophytosis prevalence in cattle is associated with intensively bred beef cattle farms as these animals are living in higher-density populations [98,99]. Pigs are another livestock species that frequently experience dermatophytosis, usually caused by *Nannizzia nana* [18] (Table 1). Dogs and goats can be infected by *N. nana* [96]. *Trichophyton equinum* is the primary dermatophyte that infects horses and rarely causes dermatophytosis in humans [97]. *Trichophyton mentagrophytes* is most commonly isolated from rodents and can be found worldwide [74,93] (Table 1). *Nannizzia persicolor* (former name *Trichophyton persicolor*) was first isolated from voles and bats and occasionally causes infections in humans [94,95] (Table 1). Zoophiles can propagate in the soil, blurring the distinction between zoophiles and geophiles [70].

### 3.3. Geophilic Dermatophytes

Geophilic dermatophytes mainly reside in soil and keratinous debris shed from animals and rarely cause infections in humans and animals [15,16]. These fungi are ecologically important as they are responsible for degrading keratin and returning the nutrients to the soil [109]. Infections caused by geophiles are generally not transmitted between hosts and are acquired from the environment [18,109]. When a geophile continues to maintain a population on particular hosts and causes more frequent infections, it would be reclassified as a zoophile [70]. The most common species to cause infection in humans and animals is *Nannizzia gypsea* (former name *Microsporum gypseum*) [16,19]. Having frequent outdoor contact with soil, particularly without protection, is a risk factor for geophilic dermatophytosis [99]. Certain professions such as farmers also have a higher risk for acquiring this infection [109].

While infections are rare, the clinical presentation of dermatophytosis caused by geophiles differs from anthropophilic and zoophilic dermatophytosis. The inflammatory response is usually more severe in these cases, and the duration of infection is generally shorter, reinforcing that host–fungus adaptation ultimately results in a diminished immune response and protracted period of replication [24]. As geophiles have not adapted to a host species, they have not been under host-specific selective pressures as much as anthropophiles [26]. Clinical signs can be similar to other dermatological diseases/disorders, resulting in difficulty in diagnosis unless culture or sequence analysis is performed [109]. *N. gypsea* can cause tinea corporis and infrequently tinea capitis in people [100] (Table 1). Geophilic species have maintained two mating types and are more likely to undergo sexual reproduction than host-adapted species [15,75,76]. It is theorized that the humid environment of soil provides a favorable condition for fruiting bodies to form, which occur during sexual reproduction. Such structures have not been isolated directly from an infected animal [70]. While habitat and other features such as reproduction differ among dermatophyte species of different classifications, the same clinical diagnostic approaches can be utilized for all dermatophytes.

## 4. Diagnostic Approaches to Dermatophytosis

An accurate diagnosis is critical for dermatophytosis to allow for early treatment and to reduce transmission to other humans or animals. When diagnosing dermatophytosis in animals, fomite carriage must be considered especially for cases without clinical signs as these cases can be positive for dermatophytosis [16]. Given the clinical presentation similarities between dermatophytosis and other skin diseases, an accurate diagnosis is also important before starting treatment [110]. Prior treatment can interfere with many diagnostic assays, leading to inaccurate results [16]. Collecting the appropriate clinical samples greatly increases diagnostic accuracy. Samples should be performed near the edge of well-defined skin lesions, as the center of the lesions may contain lowly viable to non-viable material [18]. Skin infections with poorly defined lesions should be sampled via skin scrape, covering a representative area of infection [18]. Hair samples can be collected either by plucking or using the “Mackenzie” brush technique [111]; this technique is commonly used for animals as a thorough sampling of a large area can be performed [16]. For this technique, the patient is brushed for 2–3 min or until the bristles contain a sufficient amount of hair [16]. Whole nail clippings or nail scrapings can be collected depending on the location and severity of the infection [18]. The type of clinical sample collected from the patient will influence which diagnostic approaches can be utilized as sensitivity and specificity of assays can depend on clinical sample type.

### 4.1. Direct Examination

Dermoscopy involves using a hand-held magnification tool for examining cutaneous lesions including lesions involving hair and nails [112]. Direct examination of the patient using dermoscopy is common in clinical practice, especially in human medicine [112]. As it is non-invasive, dermoscopy can be utilized for both diagnosis and for monitoring infections during treatment [112,113,114]. Modern advancements for dermoscopy include using polarized light sources and attaching the scope to a mobile device to enhance the observation of dermatological features [115]. Dermoscopy is mainly used in veterinary patients for examining hair follicles and skin [116,117]. A disadvantage for veterinary dermoscopy is compliance, as the patient needs to remain still long enough for images to be digitally captured [118] (Table 2). Consequently, the accuracy of this technique relies heavily on the skills and expertise of the examiner [118] (Table 2).

### 4.2. Wood’s Lamp

The application of a Wood’s lamp to examine for fluorescence is a commonly utilized diagnostic tool for dermatophytosis, particularly in screening animals in shelter situations [117]. This tool uses UV light (wavelength ranges between 320 and 400 nm) to detect fluorescence on skin and hair characteristic of active dermatophyte infection [119,120]. Dermatophytes that have been reported to fluoresce under the Wood’s lamp UV light are *M. canis*, *M. audouinii*, *M. ferrugineum*, *M. disortum* phenotype, *N. gypsea*, and *Trichophyton schoenleinii* [119]. The percent of *M. canis* isolates that exhibit fluorescence has been reported to range from 30 to 100% of cases [135,136,137,138,139,140]. As not all species of dermatophytes can fluoresce, a negative Wood’s lamp examination cannot rule out dermatophytosis as a diagnosis [120] (Table 2). Fluorescence can be identified even after the initiation of topical therapies including lime sulfur dips and topical shampoos [141,142,143,144,145]. Other infections and dermatological disorders such as bacterial infections, yeast infections, and pigmentary disorders can also fluoresce under Wood’s lamp, which can lead to false positives for dermatophytosis [120] (Table 2).

### 4.3. Microscopy and Histopathology

Clinical samples can be prepared and stained with various stains to enhance different fungal elements. Potassium hydroxide (KOH) can be applied to hair or skin scrapes to detect the presence of fungal elements [146,147]. While this technique is very sensitive in determining if fungi are present, it cannot discriminate between living and dead cells and cannot determine specific species [148] (Table 2). Other disadvantages of this diagnostic technique are that it requires mycological equipment and trained personnel [114] (Table 2). Lactophenol cotton blue is another stain that targets chitin in fungal cell walls, enhancing the visualization of fungal structures [149]. This stain kills the fungi, reducing potential contamination from handling the sample [149]. Mineral oil is another mounting medium for clinical samples with the advantage of not interfering with fluorescent metabolites [16,121,123]. Compared to other diagnostic methods, microscopy is relatively quick as the sample can be evaluated immediately (under 1 h) after collection [137] and has a reported false negative rate between 5 to 15% in clinical settings [18] (Table 2).

The examination of hair samples can determine if the dermatophyte species is ectothrix or endothrix as most anthropophiles are endothrix while geophiles and zoophiles are usually ectothrix [121,148]. Ectothrix means the hyphae and conidia do not invade the hair shaft and instead degrade the cuticle, while endothrix refers to fungi that invade the hair shaft [3]. Hyphal structures can also be used to distinguish the dermatophyte species. *T. mentagrophytes* has spiral hyphae, nodular bodies, or racquet hyphae [38]. *M. audouinii* is identified by pectinate bodies, which are small, hyphal projections that resemble a comb [38]. Both *T. schoenleinii* and *T. violaceum* can produce favic chandeliers, which are irregular, hyphal projections that resemble a chandelier [38].

**Figure 3:**
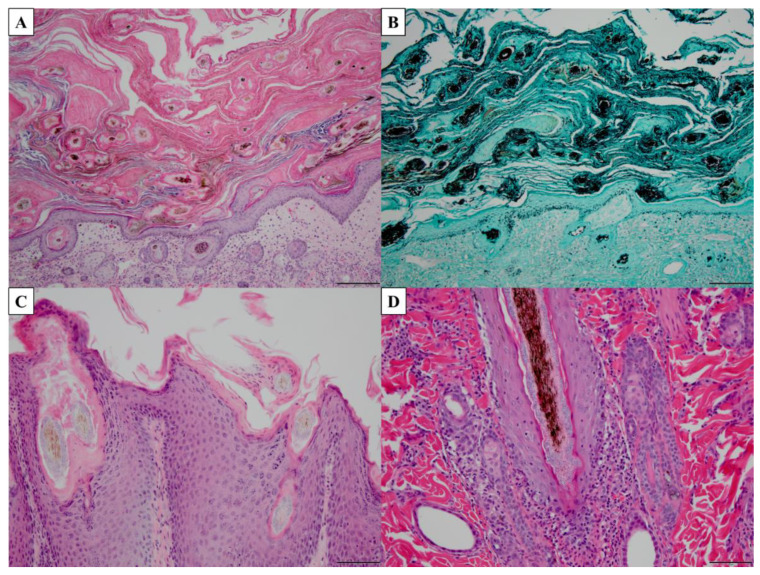
Histopathology of common histologic features of dermatophytosis. (**A**,**C**,**D**) Tissue stained with periodic acid-Schiff (PAS). (**B**) Tissue stained with Gomori’s modification of methenamine silver (GMS). (**A**) Marked hyperkeratosis (both orthokeratotic and parakeratotic), 10× magnification, scale bar = 100 µm. (**B**) Visualization of fungal hyphae, 10× magnification, scale bar = 100 µm. (**C**) Parakeratotic hyperkeratosis, acanthosis, numerous fungi associated with hair shaft, 20× magnification, scale bar = 50 µm. (**D**) Neutrophils infiltrating basal layer of hair follicle with hyphae in hair follicle lumen, 20× magnification, scale bar = 50 µm.

Histopathology is a commonly utilized technique for visualizing fungal cells in tissues. While this method is rarely used for dermatophytosis, it can be beneficial for deep dermatophyte infections as the dermatophytes have invaded the dermis or deeper tissues [11]. Histologic features observed in active dermatophytosis include parakeratosis, basket weave of the keratin layer, neutrophils in the base layers of the epidermis, spongiotic changes, eosinophils in the dermis, acanthosis or hyperkeratosis, and visualization of hyphae [150,151] (Figure 3). Stains that can be applied to the tissues to visualize the fungi include periodic acid-Schiff (PAS), Gomori’s modification of methenamine silver (GMS), and calcofluor white stain [146,150,151] (Figure 3). Given that these stains are generally not available in clinical settings and the technical skills required for histopathology, this method is infrequently used [146].

### 4.4. Fungal Culture

**Figure 4:**
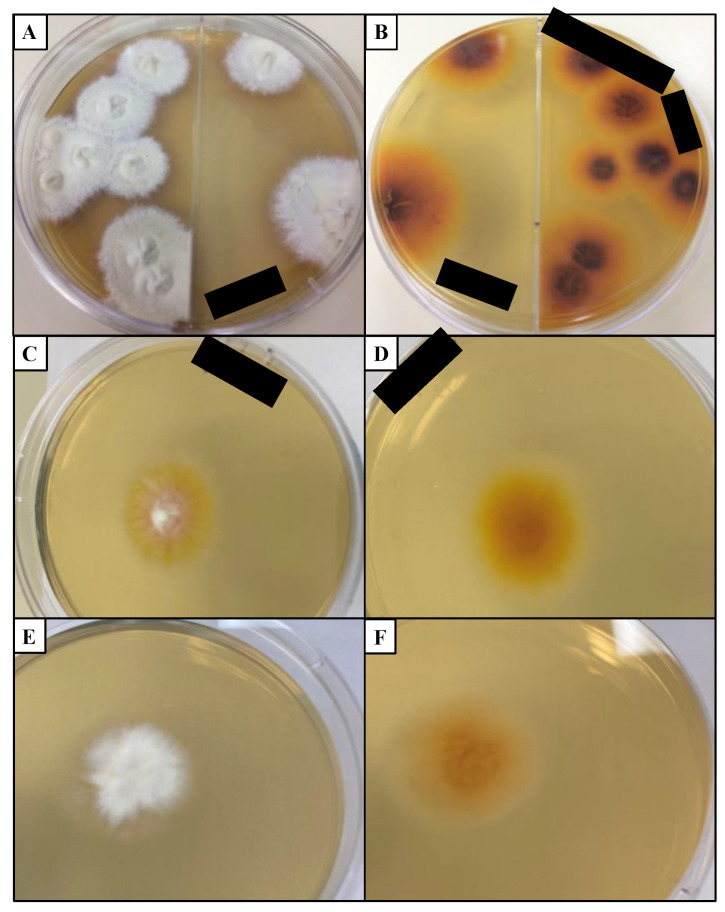
Common dermatophytes grown in Sabouraud dextrose agar (SDA) medium. (**A**) Colony surface and (**B**) colony reverse of *T. mentagrophytes* grown for 19 days isolated from domestic cat. (**C**) Colony surface and (**D**) colony reverse of *M. canis* grown for 7 days isolated from domestic cat. (**E**) Colony surface and (**F**) colony reverse of CBS 118893 *N. gypsea* grown for 7 days. Black bars: deidentifying patient information. Cultures were incubated at 20–25 °C in the dark. CBS: Westerdijk Fungal Biodiversity Institute.

Isolating and identifying dermatophytes from a clinical sample grown in a culture has been considered the “gold standard” for diagnosing dermatophytosis [122,123,124]. Dermatophyte test medium (DTM) contains phenol red, a dye that changes color when the pH increases, indicating the presence of a dermatophyte [18]. A major disadvantage of this medium is that the gross colony and microscopic morphology of the dermatophyte are altered, making it harder to determine the fungal species [147]. DTM is generally paired with Sabouraud dextrose agar (SDA) medium as the latter medium is less discriminatory and interferes less with colony morphology than DTM [152] (Figure 4). These media usually contain cycloheximide to slow the growth of non-dermatophytic fungi [18]. When trying to differentiate between *Trichophyton* species, media such as SDA with 5% salt added, vitamin free agar, Bromocresol purple milk solid glucose agar, lactritmel agar, Littman oxgall agar, and 1% peptone agar can be utilized because soil-associated *Trichophyton* species tend to be nutrient independent [74]. Rice grain slopes can be employed to distinguish *Microsporum* species because this medium induces sporulation for *M. canis* but not *M. audouinii* [74]. When grown in media, dermatophytes can produce three types of asexual conidia: macroconidia, microconidia, and arthroconidia [70]. Macroconidia have been considered to have various potential purposes from being energy sources to aiding in longevity in environments without a host [70]. These larger conidia tend to have features that deter arthropods from grazing on them, allowing for higher survival in the environment [70].

A culture is essential for tinea unguium as direct examination and microscopy can be impractical for these samples [18]. False negatives can occur due to the overgrowth of non-dermatophytic fungi, insufficient clinical material, or improper inoculation of clinical material on the culture (particularly for toothbrush samples) [16,118,153] (Table 2). False positives can occur when samples are collected from patients in contaminated environments [118] (Table 2).

Cultures for dermatophytosis are grown at room temperature (25 °C ± 5 °C) with no enhanced growth observed at the human body temperature (37 °C) [154]. In clinical practice and reference laboratories, cultures are generally grown in complete darkness [155]. However, one report did not detect a difference in dermatophyte growth when incubated with 24 h of light or darkness, 12 h of light with 12 h of darkness, or room lighting [156]. Cultures should be observed frequently for up to 4 weeks as some species are very slow growing [157] (Table 2). Determining the species growing in culture requires diagnostic expertise as pleomorphism commonly occurs [17,158] (Table 2).

### 4.5. DNA-Based Assays

Polymerase chain reaction (PCR) has increased in use as a diagnostic assay for detecting dermatophytes. PCR is a more sensitive technique than culture as it can detect fungal DNA even if the culture is negative [125,126] (Table 2). However, similar to microscopy, it cannot distinguish between living and dead fungal cells [16]. False negatives can occur due to an improper sampling technique, while false positives can occur due to nonviable fungus present on the host [16] (Table 2). A major factor that can influence the accuracy of a PCR is the DNA extraction method employed; the use of these techniques for fungal detection (versus bacteria or viruses) requires special extraction protocols that can digest the fungal cell [159]. These extra steps can involve freeze/thaw cycles, heat, or mechanical or chemical disruptions (such as beads or additional lysis buffers) [160,161].

Qualitative (conventional) PCRs for dermatophyte detection and identification generally target the internal transcriber spacer (ITS) region as this region can identify many isolates down to the species level [134]. Primers have been developed to target conserved regions of the ITS that specifically belong to dermatophytes, making it easier to identify dermatophyte-positive samples [162]. Quantitative PCRs (real-time PCR (RT-PCR)) have also been developed for dermatophyte identification from clinical samples. Using the ITS primers specifically targeting dermatophytes, RT-PCRs have been able to distinguish between dermatophyte species detected in clinical samples (hair, skin, nail) [162]. 

### 4.6. Antibody-Based Assays

The enzyme-linked immunosorbent assay (ELISA) is a common antibody–antigen-based assay that can be used for detecting various diseases [162,163,164,165,166]. Direct, indirect, sandwich, and competitive/inhibition ELISA techniques offer different advantages and disadvantages [129]. ELISA makes use of a variety of detection systems, including colorimetric/chromogenic, chemiluminescence, and fluorescent. Colorimetric is most commonly used because a standard plate reader can be utilized [167]. Sensitivity due to a low signal can be increased by optimizing reagents with a higher affinity for the target by switching the type of ELISA performed, increasing incubation times, or changing incubation temperatures [129,168]. 

ELISAs have been developed to detect dermatophytes using serum samples from clinical cases and the evaluation of antibody binding to purified *M. canis* antigen [127,128]. The *M. canis*-specific IgG antibody from cats and dogs has been detected using ELISA and had similar sensitivity to fungal cultures [127,128] (Table 2). As antibodies can persist after an infection has cleared, false positives can occur with this assay [127,128] (Table 2). Additionally, this assay requires serum, making sample collection more invasive than other diagnostic approaches (Table 2).

### 4.7. Mass Spectrometry

Matrix-assisted laser desorption/ionization time of flight mass spectrometry (MALDI-ToF MS) is gaining popularity as a diagnostic method for detecting and identifying fungal species. Filamentous fungi can have varying phenotypes, which can be detected by changes in the protein spectra [130]. While this technique is relatively fast compared to a culture, it is limited by the organism library available, making it difficult to identify novel species or rare species that are not included in the library [130] (Table 2). Additional limitations include needing an adequate sample amount, unreliable results if a sample has multiple fungal species present, spreading occurring between loaded samples, cost of initial equipment, training personnel on specialized instruments, and improper cleaning between runs [130].

Libraries have been curated for dermatophyte species with over 20 species included for analysis [131,132,133]. While this method is faster than other assays, it requires access to specialized equipment and requires that the libraries be constantly updated with name changes for the different dermatophyte species [130] (Table 2). As many of the current diagnostic approaches for dermatophyte diagnosis have major drawbacks, there is a great need for novel assays that are affordable, provide rapid results, and are user friendly.

## 5. Potential Targets of Diagnostic Assays for Dermatophytosis

Current diagnostic assays for detecting and diagnosing dermatophytosis have various disadvantages, particularly in clinical settings. One of the main challenges is distinguishing between dead and alive fungi, particularly after therapy has been implemented. A potential approach for overcoming this obstacle is to target dermatophyte-specific metabolic products because the presence of these metabolites would indicate living, metabolically active fungi. Dermatophytes produce a variety of unique metabolic products ranging from simple chemicals to complex proteins, allowing for a wide range of potential targets to be utilized for diagnostic assays [23,119,169]. Compared to other orders of fungi, dermatophyte genomes encode for a greater number of secondary metabolites including proteases [23,24]. Metabolic pathways unique to dermatophytes include those associated with keratin degradation and the production of fluorescent metabolites [23,119,169]. These pathways and metabolites will be explored below as potential targets for dermatophyte detection assays.

### 5.1. Unique Dermatophytic Keratin Metabolism, Sulfite Efflux Pump (SSU1)

Dermatophytes are unique in their ability to metabolize keratin because these proteins are, by necessity, very resistant to microbial degradation [169]. Dermatophytes secrete sulfite as well as keratinases in order to accomplish keratinolysis [169]. The *sulfite efflux pump* (SSU1) secretes sulfite to break the disulfide bonds in keratinized tissues, releasing cysteine and S-sulfocysteine (SSC) and furthering the digestion of keratin to supply fungal nutrients [169,170,171]. Cysteine, a nonessential amino acid that constitutes approximately 20% of the amino acid residues in hair, is taken up by dermatophyte fungus and internally converted to sulfite through multiple reactions [45]. Once inside the fungal cell, cysteine is oxidized to cysteine sulfinic acid by cysteine dioxygenase (Cdo1) [169]. The conversion of cysteine sulfinic acid to sulfite is theorized to occur by transamination and spontaneous decomposition, allowing for the degradation cycle to continue [171,172]. 

The SSU1 gene of a dermatophyte (*T. mentagrophytes*) has been shown to be essential for keratin degradation and clinical infection, demonstrating the potential role of this gene as a virulence factor [169]. SSU1 thus makes an attractive target for the assessment of active infection and virulence and as a factor underlying dermatophyte strain variation. Detecting metabolic products reliant on SSU1 (such as sulfite or SSC) would indicate the presence of metabolically active dermatophytes as other bacteria and yeast on the skin do not actively breakdown keratin as a nutrient source [173]. A key factor for detecting these metabolites is determining how long they can remain present on the host after the fungi has been killed to ensure false positives do not occur. Further investigation into these metabolites would be warranted for designing diagnostic assays for dermatophytosis. 

### 5.2. UV Fluorescent Metabolites

Certain dermatophyte species have been documented to fluoresce under UV light including *M. canis*, *M. audouinii, M. ferrugineum, N. gypsea,* and *Trichophyton schoenleinii* [119]. Pteridine has been reported to be the fluorescent compound produced by *M. canis* and *N. gypsea* [174,175]. Both pteridine and xanthurenic acid derivatives contribute to the fluorescence of *T. schoenleinii* [176]. These compounds produce a blue-green and yellow color when exposed to UV light, respectively [120]. Other dermatological conditions produce different colors including coral-red (bacterial infections, skin cancer), bluish-white (vitiligo and other pigmentary disorders), and brown (melasma) [120]. Detecting these fluorescent metabolites using mass spectrometry or fluorometric approaches or developing an antibody assay against the metabolites could enhance the sensitivity and specificity of diagnosing dermatophytosis compared to Wood’s lamp. Furthermore, exploring the metabolic pathways that produce these metabolites can help elucidate if all strains of the same dermatophyte species have the potential to create these compounds.

### 5.3. Dermatophyte-Specific Proteases

Proteases are a classification of enzymes that can degrade proteins into amino acids or peptides [177]. Dermatophytes express high amounts of proteases compared to other groups of fungi, particularly when exposed to keratin [23,178]. Endo- and exoproteases are produced in order to degrade keratin and downstream degradation products [24]. The proteases involved in keratin hydrolysis include groups of endoproteases and exoproteases [171]. While endoproteases break internal bonds of polypeptides, exoproteases can only target the polypeptide bonds at the N- or C-terminus [177]. The major categories of endoproteases are fungalysins, subtilisins, and neutral proteases [171,179]. The production of these proteases has been associated with increased disease severity [180]. The most important class of proteases for dermatophytes is the secreted subtilisin proteases as this family of enzymes is responsible for degrading keratin [171]. These proteins have undergone extensive expansion within dermatophytes with most species having 12 subtilisin proteases [23]. 

Subtilisin 3 (Sub3) is produced by *M. canis* and is essential for adherence to keratinized tissues during the early phases of infection [177,181,182,183,184]. Given that Sub3 expression is active during infection, detecting this protein is an indicator of metabolically active *M. canis* [169]. Sub3 produced by *M. canis* fungal cells has been detected by immunohistochemistry in domestic cat hair follicles in clinical biopsy specimens [183], suggesting that antibodies can be created to specifically target this protein. Further exploration into these potential targets could help improve dermatophyte detection with possible speciation at diagnosis. 

## 6. Introduction to *Microsporum canis*

All of the species within the *Microsporum* genus cause a significant amount of disease in both humans and animals, making this genus clinically important [14,70]. *Microsporum* includes three species, *Microsporum canis*, *Microsporum audouinii*, and *Microsporum ferrugineum*, each with genomes of approximately 23 Mbp [74]. *M. canis* is a zoophilic dermatophyte that is soil associated [14,70]. It was first described from cats and represents the earliest dermatophyte that branched from other dermatophyte species [15,17,23,185]. *M. audouinii* and *M. ferrugineum* are anthropophiles that evolved from *M. canis* [14,70]. Within the *Microsporum* genus, *M. canis* causes the majority of human infections, which are usually spread from an infected domestic cat [16]. *M. canis* used to only refer to the anamorphic form, while the teleomorph was called *Arthroderma otae* or *Nannizzia otae*; now, *M. canis* refers to both forms [15,185]. The following subsections will discuss clinically relevant topics of *M. canis* including diagnosis, prevalence, infection, and unique characteristics of *M. canis*.

### 6.1. Morphology and Laboratory Characteristics of M. canis

When grown in a culture, colonies of *M. canis* are white to cream colored with the reverse pigment ranging from golden-yellow to brownish-yellow [74]. The topography is usually flat and spreading with radial grooves and the texture is cottony to wooly [17,74]. *M. canis* is a septate, hyaline, filamentous mold that can produce different types of conidia including spindle-shaped macroconidia and microconidia [38,74]. Macroconidia have thickened cell walls, making them less digestible by arthropods that graze on conidia [70]. *M. canis* can be grown on specialized media such as lactrimel agar or rice grains to induce sporulation [74]. Unlike some *Trichophyton* species, *M. canis* does not require specialized nutrients for growth in culture [70]. 

Similar to other soil-associated dermatophytes, *M. canis* is positive for a hair perforation test by day 14 and about 80% of isolates are urease positive [17,74,186]. When grown on hair, *M. canis* is ectothrix, meaning the hyphae and conidia do not invade the hair shaft and instead degrade the cuticle [187]. The hair perforation test involves incubating the dermatophyte with hair and periodically observing the hair under a microscope to see if the hair shaft has been perforated [188]. *M. canis* isolates generally fluoresce yellow-green under UV light [16,119].

### 6.2. M. canis Habitat and Transmission 

Cats are the primary reservoir for *M. canis* with certain populations having up to 100% infection rates [137,189,190,191]. Dogs are the second most common animal reservoir, with 40–90% of dermatophytosis cases in dogs being caused by *M. canis* [108,192]. While cats and dogs frequently encounter this dermatophyte, *M. canis* is not considered a normal microbe of the host’s skin [16]. *M. canis* has rarely been isolated from horses, cattle, goats, sheep, rabbits, and pigs [93,108,193,194,195]. Transmission generally occurs by direct contact with an infected animal or contact with fomites [124,196,197]. Outbreaks frequently occur in high-density populations including animal shelters and catteries [52,123,144]. Human-to-human *M. canis* infections have been described; however, transmission wanes after a few transmission events [196,198,199]. Given the high transmissibility of *M. canis*, it has been able to spread worldwide [1,74].

### 6.3. M. canis Distribution

*M. canis* is distributed worldwide, although prevalence varies among countries [1,74]. *M. canis* has been found to be the leading cause of tinea capitis infection in Great Britain, Ireland, Western Europe, Spain, Greece, Kuwait, Hong Kong, Malaysia, Australia, New Zealand, the USA, Canada, Venezuela, Brazil, Uruguay, Argentina, Chile, Algeria, Sudan, and South Africa [200,201,202,203,204,205,206,207,208,209,210,211,212,213]. *M. canis* is also the primary agent for tinea corporis in Australia, New Zealand, Brazil, Uruguay, and South Africa [203,204,206,212]. Over the past several decades, infections due to *M. canis* have decreased due to various factors such as stray animal management [51,214].

### 6.4. M. canis Mating Types

As noted above, dermatophytes can undergo sexual or asexual reproduction depending on access to a compatible mating partner [75,215]. The two mating types of dermatophytes are the high-mobility group (HMG) and alpha-box genes, respectively. Mating-type genes are also referred to as MAT1-1 (for alpha-box) and MAT1-2 (for HMG) [75,215,216]. There have been reports of two *M. canis* mating types; however, the positive type has only been isolated from Japan [185,217,218]. It has been hypothesized that *M. canis* is becoming more reliant on asexual reproduction given the lack of MAT1-2 identification [37].

### 6.5. M. canis-Associated Clinical Disease

After keratinized tissue is exposed to *M. canis* arthroconidia, lesions generally begin appearing 1 to 3 weeks later [50]. *M. canis* infections in humans have been shown to be more inflammatory than anthropophilic *M. audouinii* infections, suggesting that *M. canis* has not adapted to human hosts [17]. Clinical signs can range from mild scaling and alopecia to severe inflammation with pustules and invasion of the dermis via the hair follicles, developing into Majocchi’s granuloma [32,219,220]. *M. canis* generally causes dermatophytosis in humans as tinea corporis and tinea capitis [18]. Tinea unguium, or infection of the nails, due to *M. canis* is rare in humans [74].

Cats with dermatophytosis generally present with mild circular alopecia and scaling [124]. Pruritus and miliary dermatitis can be variable upon presentation [16]. Dermatophytosis in cats is caused by *M. canis* in 90% to 100% of cases depending on the geographic region [108,153,218]. *M. canis* has been shown to elicit a weaker immune response in cats compared to other dermatophyte species, suggesting cats are the primary host [99,221]. *M. canis* infections in cats are less susceptible to treatment than other dermatophytes [158]. Treatment is implemented to reduce the spreading of the fungi and reduce the length of infection [222]. Clinical disease generally resolves between 7 to 17 weeks post exposure to arthroconidia [145,223].

## 7. Conclusions

Numerous dermatophyte species that infect humans and animals have recently been renamed, potentially impacting diagnosis and treatment plans. A variety of diagnostic methods have been developed for dermatophytosis with fungal culture still being considered the “gold standard”. Novel approaches for improving diagnostics include investigating assays based on fungal metabolites (sulfite metabolism, UV fluorescent metabolites, and proteases).

*M. canis* is a clinically important dermatophyte as it is a common pathogen in human and veterinary medicine and represents the most common zoonotic dermatophyte. This agent causes dermatophytosis in cats and tinea capitis and tinea corporis in humans and appears to be adapting to the feline host with the loss of one of its mating types (MAT1-2). Current diagnostic assays can identify *M. canis* and can distinguish it from other *Microsporum* species. Further investigations of the genetics and metabolism of *M. canis* (and other dermatophytes) are warranted to develop novel, rapid, and inexpensive diagnostic tests and new therapies for dermatophytosis of animals and humans.

## Figures and Tables

**Table 1:** Classification of most common dermatophytes causing infections in humans and animals. Infection nomenclature is based on infection in primary host.

Classification	Species	Primary Host/Habitat	Main Types of Infection	Geographical Distribution	Reference
Anthropophilic	*Trichophyton rubrum*	Humans	Tinea pedis, tinea unguium, tinea cruris, tinea faciei, tinea corporis, tinea manuum, tinea barbae	Worldwide	[1,18,24,25,74,86,87,89]
	*Trichophyton tonsurans*	Humans	Tinea capitis, tinea corporis, tinea faciei	Worldwide	[24,74,79,86]
	*Epidermophyton floccosum*	Humans	Tinea cruris	Worldwide	[74]
	*Trichophyton digitale*	Humans	Tinea pedis	Worldwide	[74,92]
	*Trichophyton schoenleinii*	Humans	Tinea capitis favosa	Asia, Europe, Africa	[74,81]
Zoophilic	*Microsporum canis*	Cats	Ringworm	Worldwide	[16,74,93]
	*Nannizzia persicolor* (former name *Arthroderma persicolor*)	Voles, bats	Ringworm	Africa, Australia, Europe, North America	[74,94,95]
	*Nannizzia nana* (former name *Microsporum nanum*)	Pigs	Ringworm	Worldwide	[18,74,96]
	*Trichophyton equinum*	Horses	Ringworm	Worldwide	[74,97]
	*Trichophyton mentagrophytes* (former name *Arthroderma vanbreuseghemii*)	Mice, guinea pigs	Ringworm	Worldwide	[74,93]
	*Trichophyton verrucosum*	Cattle	Ringworm	Worldwide	[74,98,99]
Geophilic	*Nannizzia gypsea* (former name *Microsporum gypseum*)	Soil	Ringworm (animals), tinea capitis/tinea corporis (humans)	Worldwide	[16,19,74,100]

**Table 2:** Comparison of diagnostic methods for detecting dermatophytes.

Diagnostic Method	Advantages	Disadvantages	Time to Results	Reference
Direct examination	Non-invasive Low cost	Unable to determine species	Minutes	[116,117]
Wood’s lamp	Non-invasive Low cost	Not all species fluoresce	Minutes	[119,120]
Microscopy	Can detect unique features of species Low cost	Unable to distinguish dead and alive fungi	Minutes	[38,121]
Culture	Low cost Easy to perform Can distinguish between species	Requires expertise to determine species Can be contaminated by saprophytes	Days–Weeks	[122,123,124]
PCR	Highly sensitive Can distinguish between species	Unable to distinguish dead and alive fungi	Hours–Days	[16,125,126]
ELISA	Highly specific	False positives due to past infections	Hours–Days	[127,128,129]
MALDI-ToF	Highly sensitive Can distinguish between species	Only detect species in library	Minutes–Hours	[130,131,132,133]
Genetic analysis	Can distinguish between species Highly sensitive	Unable to distinguish dead and alive fungi	Hours–Days	[30,31,134]

## Data Availability

Not applicable.
